# Association Between Lactoferrin Gene Polymorphism and Dental Caries in Saudi Children: A Case-Control Study

**DOI:** 10.7759/cureus.82555

**Published:** 2025-04-19

**Authors:** Narmin M Helal, Amina Bagher

**Affiliations:** 1 Department of Pediatric Dentistry, Faculty of Dentistry, King Abdulaziz University, Jeddah, SAU; 2 Department of Pharmacology and Toxicology, Faculty of Pharmacy, King Abdulaziz University, Jeddah, SAU

**Keywords:** dental caries, genotype, lactoferrin, lactotransferrin, oral cavity, pediatric patients, polymerase chain reaction (pcr), polymerase chain reaction test, single nucleotide polymorphism

## Abstract

Objective

Lactoferrin (LTF) is a salivary glycoprotein involved in the innate immune defense of the oral cavity. Polymorphisms in the *LTF* gene have been implicated in oral and systemic diseases, but limited data are available regarding their association with dental caries. This study aimed to evaluate the relationship between LTF A/G polymorphism and caries susceptibility in Saudi children.

Methods

This case-control study included 40 children aged 4-12 years treated at the pediatric dental clinics at King Abdulaziz University (KAU), Jeddah, Saudi Arabia. Participants were divided into two groups: caries-free (Decayed, Missing, and Filled Teeth (DMFT) = 0, n = 20) and caries-experienced (DMFT ≥ 1, n = 20). Salivary DNA was extracted and genotyped using polymerase chain reaction and restriction fragment length polymorphism (PCR-RFLP) analysis. Descriptive statistics were used to summarize demographic and clinical characteristics, while associations between LTF genotypes and caries status were evaluated using the chi-square test.

Results

A statistically significant association was found between the LTF genotype and dental caries status (χ² = 10.09, p = 0.006). The heterozygous AG genotype was more frequent among children with caries, while the homozygous AA genotype was more prevalent among caries-free participants. Children with the AA genotype had significantly lower odds of having caries compared to those with the AG genotype (odds ratio (OR) = 0.073, 95% confidence interval (CI): 0.012-0.431).

Conclusion

The LTF A/G polymorphism is associated with caries susceptibility in Saudi children. These findings support a potential role for salivary genetic markers in early caries risk assessment, although larger studies are needed for validation.

## Introduction

Dental caries is a complex, multifactorial, and irreversible microbial disease caused by the fermentation of carbohydrates by acid-producing bacteria. Its development is influenced by several modifiable factors, including biological, genetic, host-specific, and environmental elements [1,2]. Recent studies suggest that genetic predisposition plays a significant role in caries susceptibility, which affects approximately 35.3% of the global population [3].

Caries remains one of the most common chronic diseases in children, significantly impacting their health, well-being, and quality of life. If left untreated, early childhood caries can lead to malocclusion, impaired chewing, developmental delays, and low self-esteem [4,5].

Caries occurs when cariogenic bacteria and fermentable carbohydrates persist in the oral cavity over time. Reduced salivary flow contributes to bacterial overgrowth, increasing the risk of disease. Several biological factors, including salivary composition and flow rate, influence the cariogenicity of dental biofilms [6,7].

Saliva plays a central role in maintaining oral health. It protects teeth through mechanisms such as enamel remineralization, pH buffering, removal of food debris and microorganisms, and antimicrobial activity [8]. Factors such as low salivary flow rate or buffering capacity can compromise this defense and increase caries risk. Salivary proteins such as lysozyme, lactoperoxidase, immunoglobulins, mucins, and lactoferrin (LTF) contribute to these defenses by inhibiting the growth and adhesion of cariogenic bacteria [6].

LTF is an iron-binding glycoprotein found in various secretions, including saliva, milk, and tears. It exhibits antimicrobial, anti-inflammatory, and immunomodulatory properties [9,10]. In the oral cavity, LTF helps protect against pathogens such as *Streptococcus mutans* and* Aggregatibacter actinomycetemcomitans* [11,12]. Its antibacterial effects are mediated by the direct disruption of bacterial membranes and by depriving bacteria of iron, which is essential for their growth [10].

Genetic variation in the *LTF* gene has been linked to several systemic and oral diseases, including Parkinson's disease, breast cancer, leukemia, and aggressive periodontitis [6]. However, its role in dental caries remains less explored, and to date, no studies have investigated this association in Saudi children.

The *LTF* gene is located on chromosome 3p21 and consists of 17 exons spanning approximately 23-35 kilobases [1,13]. Single nucleotide polymorphisms (SNPs) within its coding region may alter the resulting protein's amino acid sequence, potentially affecting its antimicrobial activity. Jordan et al. reported that an SNP in the *LTF* gene was associated with aggressive periodontitis in African American patients, but not in Caucasians, suggesting a possible population-specific role of LTF variants in oral disease susceptibility [14]. Based on this evidence, the present study aimed to investigate the association between a specific A/G SNP in the *LTF* gene and dental caries risk in pediatric patients attending the dental department at King Abdulaziz University (KAU), Jeddah, Saudi Arabia.

## Materials and methods

Ethical considerations

The purpose and methods of the study were thoroughly explained to the participants and their parents or guardians. Children were enrolled after their guardians signed a written informed consent form prior to participation. The importance of the research for advancing dental care and caries prevention was also discussed. Complete confidentiality of private information was maintained, and participants were given the freedom to withdraw from the study at any time. This research complies with the Declaration of Helsinki and was approved by the King Abdulaziz University Ethical Committee (reference number: REC-046-03-22).

Study design and participants

The sample size was calculated based on established epidemiological principles and prior studies to ensure adequate power for detecting an association between LTF polymorphism and susceptibility to dental caries [15]. Using the sample size estimator for unmatched case-control studies, a two-sided confidence level of 95% and 80% power were selected. The ratio of controls to cases was 1:1, and it was assumed that 75% of the cases would be exposed. An expected odds ratio of 7 was used. Based on these parameters, G*Power estimated that the required sample size ranged from 38 to 46 participants, using Kelsey, Fleiss, and Fleiss with continuity correction.

This study included 40 healthy Saudi children aged 4-12 years who received dental treatment at the Department of Pediatric Dentistry between April and December 2022. Children were excluded if they had known allergies, were undergoing orthodontic treatment, were taking prescribed medications, had recently used anti-inflammatory agents or antibiotics (within the past six months), or had medical conditions that impair immune function. Dental caries were diagnosed using the Decayed, Missing, and Filled Teeth (DMFT) index for permanent and primary teeth. The World Health Organization (WHO) recommends using the DMFT/DMFS (Decayed, Missing, and Filled Teeth/Surfaces) indices for permanent teeth and the dmft/dmfs indices for primary teeth [16]. A consecutive sampling technique was employed. Participants were divided into two groups based on their caries experience: Group 1, with 20 children without caries (DMFT = 0), and Group 2, with 20 children with caries experience (DMFT ≥ 1).

Saliva collection

Unstimulated saliva samples were collected from all participants in a quiet room during morning hours to minimize diurnal variation [17]. Samples were obtained using Oragene®-Discover kits (DNA Genotek, Ottawa, Canada), following the manufacturer's protocol. Participants were instructed to abstain from food or drink for at least 30 minutes prior to collection. Saliva was collected up to the designated fill line to ensure adequate volume for DNA analysis. A single investigator conducted all sample collections to reduce variability. The Oragene®-Discover (OGR-500) kit was selected for its non-invasive nature, high DNA yield, room temperature stability, and compatibility with molecular techniques, including polymerase chain reaction (PCR) and genotyping. These properties make it well-suited for pediatric genetic studies.

Genotyping via polymerase chain reaction and restriction fragment length polymorphism (PCR-RFLP)

PCR-RFLP analysis was used to detect a single nucleotide polymorphism (SNP) at position 11 in the *LTF* gene, following previously published protocols [14]. A 720 base pair (bp) fragment of the gene was amplified using custom primers (forward: 5′-GAAACCACAGACCTCTGGCCAAT-3′; reverse: 5′-CTTTTGGAGGATTCCCTCTCT-3′, Macrogen, Korea). PCR reactions were performed in a 20 µL volume containing 10 µL of 2× GoTaq® Green Master Mix (Promega, Madison, WI), 100 ng genomic DNA, and 0.5 µM of each primer. Thermal cycling included an annealing step at 60°C for one minute and an extension at 72°C for one minute, followed by a final extension at 72°C for 10 minutes.

PCR products were digested using the restriction enzyme ApaLI (Alw0044I), stained with ethidium bromide, and separated by electrophoresis on a 1.5% agarose gel at 80 V for 120 minutes. Genotype identification was based on the following band patterns: AA homozygous (468 bp and 252 bp), AG heterozygous (720 bp, 468 bp, and 252 bp), and GG homozygous (720 bp only).

Statistical analysis

Data were analyzed using IBM SPSS Statistics for Windows, version 26.0 (IBM Corp., Armonk, NY). Descriptive statistics were used to summarize demographic characteristics, genotype distributions, and clinical data. Categorical variables were compared using the chi-square test. Odds ratios (ORs) with 95% confidence intervals (CIs) were calculated to evaluate the association between LTF genotypes and caries risk. Bivariate analysis was performed to assess the relationships between caries status and demographic or clinical factors, including age group, gender, DMFT levels, and genotype. A p-value of <0.05 was considered statistically significant.

## Results

The demographic characteristics of the study population are presented in Table 1. Most participants were aged 6-10 years (70%), followed by 0-5 years (17.5%) and 11-12 years (12.5%). The gender distribution was relatively balanced, with 55% male participants and 45% female participants.

**Table 1:** Demographic characteristics of the study population

Characteristic	Category	Frequency	Percentage (%)
Age (years)	4-5	7	17.50
	6-10	28	70.00
	11-12	5	12.50
Gender	Male	22	55.00
	Female	18	45.00

Genotype distributions for the LTF A/G SNP were assessed in 40 children, equally divided between caries-free (Group 1, n = 20) and caries-experienced (Group 2, n = 20) participants. In Group 1, 11 (55%) children had the homozygous AA genotype, six (30%) had the heterozygous AG genotype, and three (15%) had the homozygous GG genotype. In Group 2, two (10%) children had the AA genotype, 15 (75%) had AG, and three (15%) had GG (Table 2).

**Table 2:** LTF genotype polymorphism between groups with dental caries and without dental caries and frequencies of lactotransferrin (A/G) single nucleotide polymorphism genotype p < 0.05 indicates a significant difference in the LTF genotype polymorphism between the groups. LTF: lactotransferrin

Genotype	Group 1 (number (%))	Group 2 (number (%))	Chi-square test statistics	p-value
AA	11 (55%)	2 (10%)		
AG	6 (30%)	15 (75%)	10.0879	*0.006448
GG	3 (15%)	3 (15%)		

The AG genotype was more frequently observed among children with caries, while the AA genotype was more common in caries-free participants. A chi-square test showed a statistically significant association between LTF genotype and caries status (χ² = 10.09, p = 0.006) (Table 2). Further analysis indicated that children with the AA genotype had significantly lower odds of developing dental caries compared to those with the AG genotype (OR = 0.073, 95% CI: 0.012-0.431, p = 0.001), suggesting a potential protective role of the AA variant.

Bivariate analysis confirmed a statistically significant association between LTF genotype and caries status (p < 0.001) (Table 3). No significant associations were observed between caries status and age group (p = 0.358), gender (p = 0.057), or DMFT levels (p = 0.337).

**Table 3:** Bivariate analysis of associated factors with caries Data are presented as frequency and percentage (%). The chi-square test was used to evaluate associations between categorical variables and dental caries status. X² refers to the chi-square test statistic, indicating the degree of difference between observed and expected frequencies. p < 0.05 was considered statistically significant. ***Statistically significant atp< 0.05 DMFT: Decayed, Missing, and Filled Teeth

Variables		Caries			Total	X^2 ^value	p-value
		No (%)	Yes (%)				
Age group						2.057	0.358
0-5	2 (10.0)		5 (25.0)	7 (17.5)			
6-10	16 (80.0)		12 (60.0)	28 (70.)			
11-12	2 (10.0)		3 (15.0)	5 (12.5)			
Gender						3.636	0.057
Male	14 (70.0)		8 (40.0)	22 (55.0)			
Female	6 (30.0)		12 (60.0)	18 (45.0)			
Lactoferrin group						10.0879	<0.001***
AA	11 (55.0)		2 (10.0)	13 (32.5)			
AG	6 (30.0)		15 (75.0)	21 (52.5)			
GG	3 (15.0)		3 (15.0)	6 (15.0)			
DMFT levels						3.377	0.337
0-5 (low)	8 (42.1)		4 (20.0)	12 (30.8)			
6-10 (middle low)	2 (10.5)		6 (30.0)	8 (20.5)			
11-15 (middle high)	7 (36.8)		8 (40.0)	15 (38.5)			
16-20 (high)	2 (10.5)		2 (10.0)	4 (10.3)			

## Discussion

This study examined the association between LTF A/G polymorphism and dental caries in a pediatric population. Although previous studies have linked six LTF SNPs to periodontal disease and caries, this is the first investigation conducted in a Saudi cohort [14]. Using PCR-RFLP, three genotypes (AA, AG, and GG) were identified, consistent with earlier reports [6,18]. PCR-based methods such as RFLP and single-strand conformation polymorphism (SSCP) are widely recognized for their sensitivity in detecting point mutations and genetic variants [19].

Our findings showed that the AG genotype was more prevalent among children with caries, while the AA genotype was more common among caries-free participants. This distribution is consistent with the findings of Azevedo et al. [6], who observed distinct band migration patterns caused by an A-to-G substitution, and aligns with previously reported trends in caries-susceptible individuals. Overall, the AG genotype predominated in our sample, in agreement with the observations of Karayasheva et al. [20].

Importantly, the AA genotype appeared to be protective, particularly among children without caries, while the GG genotype was least represented across both groups. This supports prior reports suggesting that the lysine residue (AA variant) exhibits stronger antibacterial activity against *Streptococcus mutans* than the arginine variant (GG), which may account for the reduced caries susceptibility associated with AA [21].

Previous studies have explored the role of LTF in oral immunity and its association with inflammatory oral diseases. Jordan et al. demonstrated that a coding polymorphism in the N-terminal region of the *LTF* gene was associated with increased susceptibility to aggressive periodontitis in African American populations, suggesting that LTF variants may influence immune responses and biofilm dynamics [14]. Velliyagounder et al. further emphasized the antimicrobial and immunomodulatory functions of LTF in the oral cavity, particularly its roles in iron sequestration and inhibition of cariogenic bacteria [9]. Although LTF has been more extensively studied in periodontitis, these findings provided a scientific rationale for investigating its potential role in dental caries, given the shared immune and microbial mechanisms underlying both diseases. Additionally, Harris et al. highlighted the multifactorial nature of dental caries and the need to consider both biological and environmental risk factors when studying susceptible populations [22]. Genetic contributions to caries risk have also been emphasized, with specific variants proposed to influence susceptibility through salivary protein expression and host response [23].

Although salivary flow was not assessed in this study, earlier work has reported an inverse relationship between salivary flow rate and caries prevalence [6]. The AA genotype has also been associated with higher salivary flow and lower DMFT scores, suggesting a dual protective role in both salivary quantity and antimicrobial activity.

The results reinforce the concept that dental caries is a multifactorial disease involving complex interactions between genetic, biological, and environmental factors. While no significant associations were found between caries status and age, gender, or DMFT levels, genotype distribution differed significantly between groups (Table 3), indicating a potential biological influence that is independent of demographic variables.

Inconsistencies in previous literature on salivary proteins and caries susceptibility may be attributed to methodological differences, variability in individual protein expression, and complex interprotein interactions [24]. Salivary components such as lactoferrin, IgA, lysozyme, and peroxidase may act synergistically or antagonistically. For instance, IgA may modulate lactoferrin's bactericidal effects, while lysozyme and hypothiocyanite can enhance its membrane-disruptive properties [14]. However, most studies have evaluated individual salivary proteins in isolation, despite the likely presence of compensatory mechanisms within the broader protein network.

Study limitations

This study is limited by its small sample size and cross-sectional design, which restricts generalizability and precludes causal inference. Additionally, important confounding variables such as dietary habits and oral hygiene practices were not controlled and should be accounted for in future research.

Future recommendations and clinical implications

These findings provide preliminary evidence supporting a potential role for LTF polymorphisms in caries susceptibility. Although the AG genotype was more common among children with caries, the clinical utility of LTF genotyping remains exploratory. Future studies should aim to validate these associations in larger and more diverse cohorts while also investigating gene-environment interactions in greater depth. Functional studies examining the biological activity of LTF variants and their interactions with other salivary proteins may offer additional insight into host defense mechanisms in the oral cavity.

If confirmed, LTF variants could eventually be integrated into caries risk prediction models, contributing to more personalized prevention strategies in pediatric dentistry and advancing the field of precision oral healthcare.

## Conclusions

This study highlights a statistically significant association between the *LTF* gene polymorphism and dental caries susceptibility in a pediatric Saudi population. Specifically, the heterozygous AG genotype appears to be linked with an increased risk of developing dental caries, while the homozygous AA genotype may confer a protective effect. These findings suggest that genetic variations in innate salivary defense mechanisms may play a substantial role in the pathogenesis of dental caries. Given the multifactorial nature of caries, integrating genetic screening with traditional risk assessments may enhance early identification of high-risk individuals and allow for more personalized preventive strategies.
